# Retraction Note: Liposome-lentivirus for miRNA therapy with molecular mechanism study

**DOI:** 10.1186/s12951-026-05067-w

**Published:** 2026-09-15

**Authors:** Fen Sun, Huaqing Chen, Xiaoyong Dai, Yibo Hou, Jing Li, Yinghe Zhang, Laiqiang Huang, Bing Guo, Dongye Yang

**Affiliations:** 1 Institute of Animal Sciences and Veterinary Medicine, Shandong Academy of Agricultural Sciences, Jinan, 250000 China; 2 School of Life Sciences, Tsinghua University, Beijing, 100084 China; 3 State Key Laboratory of Chemical Oncogenomics, Tsinghua Shenzhen International Graduate School, Tsinghua University, Shenzhen, 518055 China; 4 Precision Medicine and Healthcare Research Center, Tsinghua-Berkeley Shenzhen Institute (TBSI), Tsinghua University, Shenzhen, 518055 Guangdong China; 5 Shenzhen Key Laboratory of Advanced Functional Carbon Materials Research and Comprehensive Application, School of Science, Harbin Institute of Technology, Shenzhen, 518055 China; 6 Shenzhen Key Laboratory of Flexible Printed Electronics Technology, Harbin Institute of Technology, Shenzhen, 518055 China; 7 Division of Gastroenterology and Hepatology, The University of Hongkong-Shenzhen Hospital, Shenzhen, China

**Retraction Note: Journal of Nanobiotechnology (2024) 22:329**

**https://doi.org/10.1186/s12951-024-02534-0**

The Editor-in-Chief has retracted this article. Concerns were raised regarding a number of figures, specifically:

- Figure 4d and 4e: the CD133 band of Figure 4d appears to overlap with the Snail band of Figure 4e;
- Figure 4a and 7a: the c-MYC band of Figure 4a appears to overlap with the p-GSK-3B S9 band of Figure 7a when rotated;
- Figure 6b and 6i: the β-actin bands between the two figures appear to overlap when rotated;
- Figure 2e and 6d: the D0 and D7 Control panels of these figures appear to overlap;
- Figure 2a, 2d, 2i, and 5e: the β-actin bands of these figures appear to overlap;
- Figure 4a, 4e, and 7b: the β-actin bands of these figures appear to overlap;
- Figure 7d, 7e, and 7j: the β-actin bands of these figures appear to overlap.

The authors provided some source data on request from the Publisher, but this was insufficient to address the concerns raised. The Editor-in-Chief therefore no longer has confidence in the reliability of the data reported in the article.

Fen Sun, Huaqing Chen, Xiaoyong Dai, Jing Li, and Dongye Yang do not agree to this retraction. Yibo Hou, Yinghe Zhang, Laiqiang Huang, and Bing Guo have not responded to any correspondence regarding this retraction. 

